# Pathological Processes Among Content Creators on Social Media: Scoping Review

**DOI:** 10.2196/76708

**Published:** 2025-09-05

**Authors:** Sergio Martinez-Aguirre, Javier Sanz-Valero, Elena Ronda-Pérez

**Affiliations:** 1 Doctoral Program in Health Sciences, University of Alicante, Alicante, Spain; 2 National School of Occupational Medicine, Carlos III Health Institute, Monforte de Lemos Street, 5, Madrid, 28029, Spain, 34 918224056; 3 Public Health Research Group, Department of Community Nursing, Preventive Medicine and Public Health and History of Science, University of Alicante, CIBER of Epidemiology and Public Health, Alicante, Spain

**Keywords:** pathological processes, social media, content creator, psychological phenomena, postural balance

## Abstract

**Background:**

Content creators (CCs), like any other worker, are exposed to various occupational hazards that can affect their physical, mental, and social well-being, with psychosocial and ergonomic risks being particularly relevant. The combination of prolonged work hours, sedentary lifestyles, excessive public scrutiny, and often job insecurity and unpredictability (manifested as continuous connectivity and anticipation of sporadic tasks) presents a significant risk for the development of health issues.

**Objective:**

This study reviews the scientific literature to identify the potential pathological processes affecting CCs on social media.

**Methods:**

The scoping review method was used. Data were obtained from the following bibliographic databases: MEDLINE (via PubMed), Embase, Cochrane Library, PsycINFO, Scopus, Web of Science, and Virtual Health Library. The terms used as descriptors and in the title and abstract fields were “Content Creator” and “Pathologic Processes.” The search was conducted in May 2024. Agreement between authors for paper selection was measured using the Cohen κ coefficient. The documentary quality of the papers was assessed using the STROBE (Strengthening the Reporting of Observational Studies in Epidemiology) questionnaire, and the level of evidence and recommendation grade were determined according to the Scottish Intercollegiate Guidelines Network recommendations. Bias was evaluated using the Risk of Bias in Non-randomized Studies of Exposures (ROBINS-E) tool.

**Results:**

Of the 1522 references retrieved, 6 papers were selected based on the inclusion and exclusion criteria. Of the 6 studies reviewed, 3 were exclusively focused on a single gender. The agreement on the relevance of the selected studies, calculated using the κ index, was 84.9% (*P*<.01). The study population ranged from a minimum of 6 to a maximum of 1544 participants. The STROBE scores ranged from 81.3% to 96.8%, with a median of 14.9% (IQR 2.1). According to the Scottish Intercollegiate Guidelines Network criteria, this review provided evidence level 2++ with a recommendation grade of B. ROBINS-E highlighted a higher number of biases in Domains 5, 6, and 7. All interventions were based on interviews, either conducted online or via email. Participant activities, as documented in the respective studies, comprised influencer roles (n=2), blogging (n=2), YouTube content creation (n=1), and live streaming (n=1). The design of the reviewed works comprised 4 qualitative studies and 2 mixed methods (qualitative and quantitative) studies. The reported health impacts were diverse, comprising burnout (n=2), anxiety (n=1), co-occurring anxiety and depression (n=1), eating disorder (n=1), chronic pain (n=1), and unspecified mental health issues (n=1). All studies highlighted the necessity for further investigation into potential pathological processes among CCs engaged in social media activities.

**Conclusions:**

It was found that the most affected area was mental health, as observed in nearly all the reviewed studies. Despite the extensive documentation of mental health impact, it is necessary to identify the risk factors associated with the pathological processes of CCs to prevent the signs and symptoms identified in this literature review.

## Introduction

The rise of social media is primarily supported by the creation of content that appeals to the public. These social media platforms have become not only a means of social entertainment but also a commercial medium where brands offer their products and services through individuals with a significant ability to influence a broad audience.

In this framework, the role of content creation (CC) stands out as a new form of employment. The emergence of this labor role, popularly known as the “influencer,” led the European Union to enact the Digital Services Act, which introduced regulations for digital platforms and CCs to combat illegal content and misinformation [1]. This regulation prompted the Spanish government to introduce specific legislation that established the rights and obligations for those engaging in this profession [2].

Moreover, Spain does not currently possess specific legislation governing the working hours of CCs, nor is there any European regulation directly addressing the working time (broadcast duration) of CCs.

Thus, the exercise of this activity, as indicated by a study from the University of Valencia [3], is based on the economic performance derived from the advertising impact it has on social media. In other words, it connects commercial brands with their target audience through their ability to influence a community. Collaboration with CCs is now part of the global strategy of brands [4]. To achieve this, CCs must devote much of their day not only to content creation but also to promoting their work and engaging with sponsoring companies.

In the context of their professional activity, like any other worker, CCs are exposed to various occupational risks that can affect their physical, mental, and social well-being [5]. Particularly relevant are psychosocial and ergonomic risks. Psychosocial risks are related to factors arising from the interaction between work, individuals, and the social context: stress, pressure to maintain a constant presence on digital platforms, or public exposure can lead to anxiety or emotional disorders. Regarding ergonomic risks, these relate to the organization and conditions of the work environment: the use of data display screens, forced postures, or repetitive movements. Identifying, evaluating, and preventing these risks must be the foundation of this labor sector to avoid future health problems in CCs.

Although the negative health implications of social media engagement are recognized, there is limited understanding regarding the precise role social media plays in the development of associated pathological conditions [6]. Thus, this cocktail of marathon workdays, sedentary behavior, public overexposure, and, in many cases, job insecurity and uncertainty (being constantly connected and awaiting tasks without predictability) results in health problems, ranging from mild to severe [7].

Similarly, CCs have been associated with components of addiction (prominence, tolerance, mood modification, relapses, withdrawal, and conflict). However, Peng and Liao [8] have critiqued the ability to differentiate between problematic and highly active CCs. Recently, a novel media phenomenon termed “pathostreaming” has gained prominence. “Pathostreaming” refers to sharing misleading or harmful content (eg, obscenity and hate speech) online, alongside the propagation of hate speech across various digital platforms. The distinct characteristics of “pathostreaming” have led to the emergence of new media entities: “pathostreamers,” “pathoinfluencers,” and “pathousers.” A critical area of ongoing research focuses on the mental health of these individuals and their influence on their followers [9].

There is no doubt that work through digital platforms is growing in importance; however, it also presents significant challenges for the health and safety of its workers. Specifically, all the risks involved in the activities will be present (transport, cleaning, workplace adaptation, home-based work, etc), but these are exacerbated by the unique characteristics of work on platforms [10,11].

CCs are often expected to produce quality work while managing their own stress. The pressure of the creative process, combined with the challenge of meeting deadlines, can lead to burnout. One of the main forms of digital content creation is the live streaming of video games. The study noted that video game players and those engaged in electronic sports were at a higher risk of developing numerous chronic diseases and experiencing increased mortality, highlighting a concerning reality in this research [12].

At the same time, participation on this Web 2.0 platform, with prolonged exposure as an idealized image, was correlated with depressive symptoms, self-esteem problems, anxiety, and body dissatisfaction [13]. Furthermore, this overexposure may occasionally result in the reception of hate speech. The experience of being targeted by hate speech is distressing for content creators (CCs); this sense of attack can diminish their self-perception and self-esteem, thereby compromising their well-being [14]. In this line, Fung et al [15] conducted a systematic review that highlighted the importance of social media in public health issues. They emphasized how the generated content influenced both the well-being of creators and viewers.

All of the above justifies the need for this review. In recent years, social media has experienced a remarkable surge, giving rise to new forms of employment. Among these, CCs stand out as a rapidly growing and increasingly influential group across various sectors. This professional field has evolved and diversified, attracting a significant portion of the active workforce, particularly among younger generations.

Therefore, the central aim of this study is to critically analyze the available scientific bibliography, seeking to understand the potential pathological processes derived from CC within the realm of social networks and the interventions undertaken, with the ultimate aim of establishing the nature of their causal link.

## Methods

### Design

This is a descriptive cross-sectional study and a critical analysis of studies retrieved through a systematic technique. The structure of this review followed the guidelines of the PRISMA (Preferred Reporting Items for Systematic Reviews and Meta-Analyses) [16] and the methodological framework proposed by Arksey and O’Malley [17] for scoping reviews.

### Protocol Registration

The protocol of this review was registered in the institutional repository REPISALUD of the Instituto de Salud Carlos III.

### Study Population

In this review, a CC was defined as an individual responsible for generating and distributing diverse forms of material (including text, video, audio, and images) across digital platforms. Their primary aims include entertaining, informing, educating, or fostering a connection with an audience. This creative work can be performed on any web-based social media platform.

### Data Collection Source

Data were obtained via direct consultation of the following bibliographic databases in the field of health sciences: MEDLINE (via PubMed), Embase, Cochrane Library, PsycINFO, Scopus, Web of Science, and Virtual Health Library.

### Information Processing

To define the search terms, the Thesaurus of Descriptores de Ciencias de la Salud, developed by the Latin American and Caribbean Center on Health Sciences Information, was consulted along with its equivalence to Medical Subject Headings from the US National Library of Medicine.

From the hierarchical study of both thesauri and their indexing sheets (entry terms), the following search equations were deemed appropriate:

Equation 1: CCs on social media (Web 2.0).

“Content Creator*”[Title/Abstract] OR “Influencer*”[Title/Abstract] OR “Streamer*”[Title/Abstract] OR “Gamer*”[Title/Abstract] OR “Youtuber*”[Title/Abstract] OR “TikToker*”[Title/Abstract] OR “Instagramer*”[Title/Abstract] OR “Twitcher*”[Title/Abstract] OR “Blogger*”[Title/Abstract] OR “Vloggers*”[Title/Abstract] OR “Podcaster*”[Title/Abstract] OR “Content Strategist”[Title/Abstract]

Equation 2: pathological processes—abnormal forms and mechanisms involved in dysfunctions of tissues and organs.

“Pathologic Processes”[Mesh] OR “Pathologic Processes”[Title/Abstract] OR “Pathological Process”[Title/Abstract] OR “Disease*”[Title/Abstract] OR “Syndrome*”[Title/Abstract] OR “Symptom*”[Title/Abstract] OR “Illness”[Title/Abstract]

The final search equation was developed for MEDLINE (via PubMed) by combining the two proposed equations using Boolean logic (Equation 1 and Equation 2).

This strategy was then adapted to the characteristics of each of the other consulted databases, and the search was conducted from the earliest available date up to May 2024. In addition, a complementary search strategy was implemented to minimize the possibility of publication bias by manually searching the bibliographies of the papers selected for the review. Furthermore, experts in the study topic were contacted to determine the potential existence of gray literature (materials and research produced by organizations outside of traditional commercial or academic publications and disseminated through alternative channels). These experts were not involved in the delineation of the research scope, the refinement of the research question, the selection of studies, or the interpretation of the findings.

### Final Selection of Papers

The selection of papers for review and critical analysis was informed by the criteria outlined in Textbox 1.

Textbox 1. **Inclusion and exclusion criteria adopted for the review.**

**Inclusion criteria:**

- Original paper published in a peer-reviewed journal.
- The language must be English, Spanish, or Portuguese.
- The complete paper is available for review.
- Participants aged 13 years and older (minimum legal age for engagement in the activity).

**Exclusion criteria:**

- Studies that do not address the objectives of the review (eg, content creator [CC] and pathological processes).
- Studies conducted exclusively with participants under 13 years [2].

The selection of relevant papers was performed by two authors of this review (SMA and JSV). To validate the inclusion of papers, the agreement rating between the authors had to be above 0.60 [18]. Any disagreements were resolved through consensus among all authors of the review.

### Document Quality, Evidence Level, and Recommendation Grade

The structural validity of the papers was evaluated using the STROBE (Strengthening the Reporting of Observational Studies in Epidemiology) guidelines [19], which contain a list of 22 essential aspects that must be described in each paper. For each selected paper, one point was assigned for each item present. If an item contained several subcategories, these were independently evaluated, with the same value given to each subcategory, and then an average was calculated (this average was the result for that item), ensuring that the total score for each item did not exceed one point.

To determine the level of evidence and its recommendation grade, the recommendations of the Scottish Intercollegiate Guidelines Network (SIGN) Grading Review Group [20] were used.

### Bias Study

The risk of bias in exposure effect studies was assessed using the Risk of Bias in Non-randomized Studies of Exposures (ROBINS-E) tool [21]. This tool classifies biases into 7 dimensions, rating each one on four possible interpretations: low risk (little or no concern about bias); some concerns (there is some concern about bias in this domain, though it is unclear whether a risk exists); high (the study has some significant issues in this domain, leading to a high risk of bias); and very high (the study is highly problematic in this domain. The study’s characteristics lead to a very high risk of bias.

### Data Extraction

Data accuracy control was performed using double-entry tables that allowed for the detection of deviations and their correction through reconsulting the original data. Duplicate records were excluded using the cross-platform ZOTERO program (George Mason University), a reference management tool developed by the Center for History and New Media at George Mason University.

### Variables Under Study

The papers were grouped based on the variables under study to systematize and facilitate the understanding of the results. The following data were extracted: first author, year of publication, design, population (CC activity and age), disease, platform or location, time frame, instrument, and health issue.

### Data Analysis

The data related to information retrieval were presented in terms of frequency and percentage. The agreement for paper selection by the two authors was measured using Cohen κ coefficient, with an agreement considered adequate when the value was above 60% (good or very good agreement strength).

To determine the currency or obsolescence of the selected papers, the Burton-Kebler (BK) semiperiod was calculated—defined as the median age based on the temporal range analyzed—and the Price Index (PI), which represents the percentage of papers published within the past 5 years.

STROBE questionnaire scores were analyzed using the median, maximum, and minimum. The evolution of these scores, in relation to the years of publication, was obtained through regression analysis (*R*^2^).

For statistical analyses, SPSS (version 29; IBM Corp) for Windows was used. The level of significance used was *α*=.05.

### Ethical Considerations

All data were obtained from papers accepted for review. Therefore, in accordance with Law 14/2007 on biomedical research, ethical committee approval was not required due to the use of secondary data [22].

## Results

### Overview

By applying the search criteria, a total of 1522 references were retrieved: 200 (13.1%) from MEDLINE (via PubMed), 61 (4.0%) from Embase, 148 (9.7%) from Cochrane Library, 95 (6.2%) from PsycINFO, 519 (34.1%) from Scopus, 344 (22.6%) from Web of Science, and 155 (10.2%) from Virtual Health Library. The consultation of bibliographies of selected papers and expert consultation did not yield any new documents. The entire paper selection process can be referenced.

**Figure 1.**
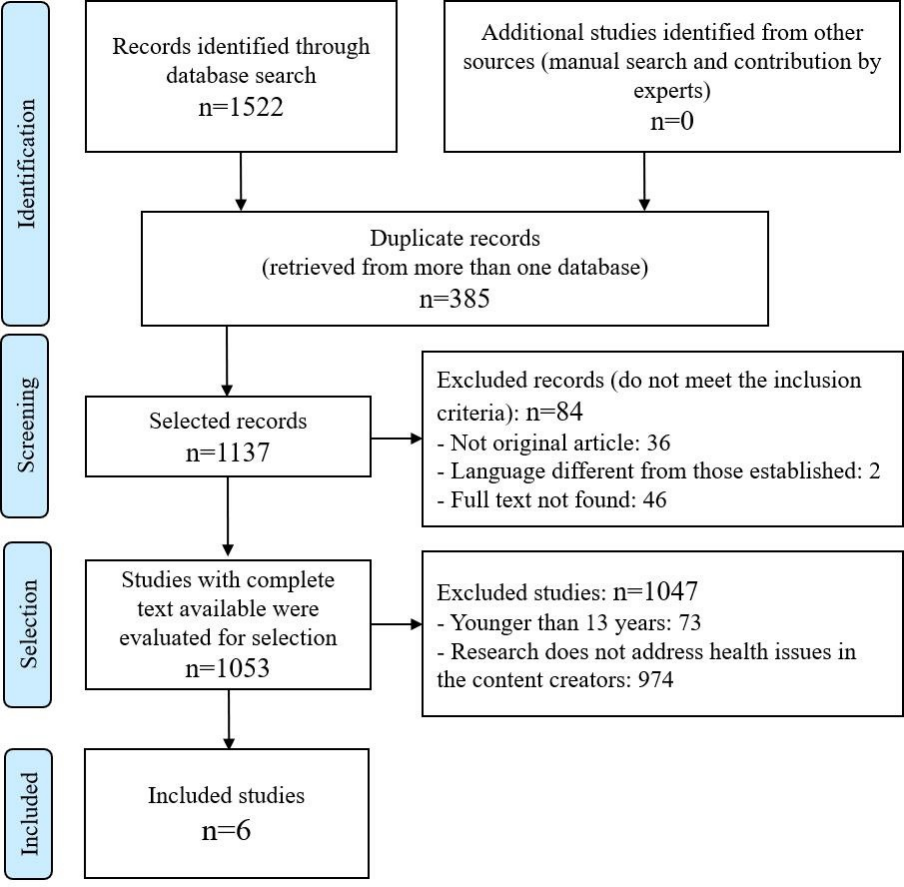
Identification and selection of studies in accordance with the PRISMA (Preferred Reporting Items for Systematic Reviews and Meta-Analyses) guidelines.

After eliminating 385 duplicate records and applying the inclusion and exclusion criteria (Figure 1), 6 documents [23-28] were selected for review and critical analysis; refer to Table 1.

**Table 1.** Selected studies for review on pathological processes among content creators on social networks.

Author	Design	Activity	Population (n; gender; age)	Disease	Platform or location	Time frame	Instrument	Health issue
Zsila et al [23]	Qualitative and quantitative	Influencer	Study 1: n=1544; M/W^a^=1375/169; 14-53 years Studies 2 and 3: n=525; M/W=427/98; 18-56 years	Depression and anxiety	Twitch	Study 1: spring 2021 Study 2: 2021‐2022 Study 3: 2021‐2022	Study 1: online questionnaire on gaming disorder (DSM-5^b^ criteria) Study 2: 10-item Need for Belonging Scale. The participants’ need for group dependency was evaluated. Study 3: following the same approach as Study 2, but the focus was on aspirational identification and parasocial relationships	Study 1: internet gaming disorder, inadequate sleep, and symptoms of depression and anxiety Study 2: high scores were obtained, indicating the need for belonging Study 3: investigates the type of contact with the streamer, parasocial relationship bonds, identification of desires, and the need for belonging as predictors of financial support to a favorite streamer
Niederauer and Maggi [24]	Qualitative	YouTuber	n=6; M/W=6/0; 18-40 years	Mental health (burnout)	YouTube	Not specified	Semistructured interviews addressing subjective questions about the digital influencer on the YouTube platform	Exhaustion, discomfort, pressure, distress or anxiety, and burnout
Gandhi et al [25]	Qualitative	Streamer	n=25; M/W/O=16/8/1; 18-39 years	Mental health (unspecified)	Twitch	2019	Semistructured interviews with an average duration of 59 minutes (SD: 25.5); $20 per interview	Mental health issues (no specific symptoms are outlined); the study focuses on how streamers, viewers, or both address these issues during live streams
Tran et al [26]	Qualitative	Influencer	n=9; M/W=0/9; 21-40 years	Burnout	YouTube	Not specified	20-question interview distributed via email (Jacobsen Framework, theoretical model for the study of esthetics psychology)	Burnout (feelings of guilt related to inconsistent work, delayed video posts, creative blocks, and failure to keep social media platforms updated) Mood regulation and self-esteem Anxiety
Yeshua-Katz and Martins [27]	Qualitative	Blogger	n=33; M/W=0/33; 15-33 years	Eating disorder	Blog	Not specified	Semistructured interview with three sections of questions: First section: bloggers’ experiences with their eating disorder Second section: focused on their reasons for blogging about their disorder Third section: questions about the perceived benefits and disadvantages of pro-ana blogging	Eating disorder (anorexia nervosa) Anxiety
Ressler et al [28]	Qualitative and quantitative	Blogger	n=230; M/W=172/39; 18-75 years	Chronic pain	Blog	May 5 to July 11, 2011	Online open-ended survey with 34 items	Chronic pain diseases Therapeutic potential of blogging (the act of posting about the pathology they endure—in this case, chronic pain diseases—allows individuals to alleviate the emotional burden associated with it)

a M/W/O: man/woman/other (nonbinary).

b DSM-5: *Diagnostic and Statistical Manual of Mental Disorders, Fifth Edition*.

The agreement on the relevance of the selected studies between the evaluators, calculated using the κ index, was 84.9% (*P*<.01).

The total retrieved papers showed an obsolescence rate, according to the BK semiperiod, of 3 years, with a PI of 71.40% (7/10 papers were published in the last 4 years). Meanwhile, the papers selected for review showed a BK semiperiod of 3.5 years, with a PI of 66.7%.

When evaluating the document correction of the reviewed papers using the STROBE questionnaire, the scores ranged from a minimum of 13.0 (81.3% compliance) to a maximum of 15.5 (96.8% compliance), with a median of 14.9 (Table 2). A very low, nonsignificant direct linear trend over time was observed (*R*²=0.02; *P*=.80).

**Table 2.** Analysis of the methodological quality of the studies according to the 22 evaluation points of the Strengthening the Reporting of Observational Studies in Epidemiology guidelines.

Paper	Score of the points in the questionnaire^a^																						
	1	2	3	4	5	6	7	8	9	10	11	12	13	14	15	16	17	18	19	20	21	22	Value, n (%^b^)
Zsila et al [23]	1	1	1	1	1	1	1	1	N/A	1	N/A	N/A	1	0.5	N/A	N/A	N/A	1	1	1	1	0.5	15.0 (93.8)
Niederauer et al [24]	0.5	1	1	1	1	1	1	0	N/A	1	N/A	N/A	1	1	N/A	N/A	N/A	1	1	1	1	1	14.5 (90.6)
Gandhi et al [25]	1	1	1	1	1	1	1	1	N/A	1	N/A	N/A	1	1	N/A	N/A	N/A	1	1	1	1	0.5	15.5 (96.8)
Tran et al [26]	0.5	1	1	1	1	1	1	1	N/A	1	N/A	N/A	0.3	1	N/A	N/A	N/A	1	1	1	1	1	14.8 (92.5)
Yeshua-Katz et al [27]	0.5	1	1	0	1	1	1	1	N/A	1	N/A	N/A	0.5	1	N/A	N/A	N/A	1	1	1	1	0	13.0 (81.3)
Ressler et al [28]	0.5	1	1	1	1	1	1	1	N/A	1	N/A	N/A	1	1	N/A	N/A	N/A	1	1	1	1	1	15.5 (96.8)

a 0: does not meet the item or any of its parts; 1: fully meets the item; 0 to 1: partially meets the item; and NA: does not apply.

b Percentage of compliance for the total items, excluding those that do not apply (N/A).

According to SIGN criteria, this review presented an evidence level of 2++ (systematic reviews with a high probability of a causal relationship) with a recommendation grade of B (a body of evidence including studies directly applicable to the target population and showing overall consistency of results).

The risk of bias in the analyzed studies and the evaluation of the domains included in the ROBINS-E tool are summarized in Table 3, where a higher number of biases were observed in older papers, particularly in Domains 5, 6, and 7. This indicates that these studies have difficulty in appropriately measuring the results.

**Table 3.** Evaluation of the reviewed papers according to the bias domains included in the Risk of Bias in Non-randomized Studies of Exposures tool.

Domain	Zsila et al [23]	Niederauer et al [24]	Gandhi et al [25]	Tran et al [26]	Yeshua-Katz et al [27]	Ressler et al [28]
Domain 1^a^	Some concerns	Low	Low	Low	Low	Low
Domain 2^b^	Low	Low	Some concerns	Some concerns	Low	Some concerns
Domain 3^c^	Low	Low	Low	Low	Some concerns	Low
Domain 4^d^	N/A^e^	N/A	N/A	N/A	N/A	N/A
Domain 5^f^	Some concerns	Very high	High	Very high	High	Some concerns
Domain 6^g^	High	High	High	Very high	High	High
Domain 7^h^	High	High	High	High	High	High

a Domain 1: due to confounding.

b Domain 2: arising from exposure measurement.

c Domain 3: participant selection in the study.

d Domain 4: due to interventions after exposure.

e N/A: not applicable.

f Domain 5: due to missing data.

g Domain 6: arising from outcome measurement.

h Domain 7: due to the selection of the reported outcome.

The study population ranged from a minimum of 6 participants [24] to a maximum of 1544 participants [23]. Three of the 6 studies focused on a single gender: Niederauer and Maggi [24] included only men, while Tran et al [26] and Yeshua-Katz and Martins [27] included only women. Gandhi et al [25] was the only study that identified gender differences. Two studies noted that the population initially presented eating disorders [27] and chronic pain [28], while the other studies did not report any specific pathology in the studied population.

The activities performed by the individuals included in the various studies were: influencers [23,26], YouTubers [24], streamers [25], and bloggers [27,28].

Although this review focused on adults, as it investigated CC as a professional activity, two studies included minors: the studies by Zsila et al [23] and Yeshua-Katz et al [27]. Only the study by Ressler et al [28] included individuals older than 65 years.

### Tools Used

All tools were shared either online or via email. The study by Zsila et al [23] was mixed (qualitative-quantitative). It analyzed qualitative aspects and established quantitative scales or values through 3 sections. It examined the problematic gaming behavior pattern, characterized by an increasing priority given to gaming over other activities, to the point where gaming takes precedence over daily interests and activities (gaming disorder). It also assessed the sense of trust, intimacy, and acceptance within a group, whether family, friends, or work (Need for Belonging Scale), and considered the potential financial support based on the second point.

Niederauer and Maggi [24] conducted an exploratory qualitative study using semistructured interviews. The methodology involved content analysis of participants’ responses to identify patterns and generate thematic categories.

Gandhi et al [25] used a qualitative methodology with semistructured interviews to explore how CCs address mental health topics.

Tran et al [26] conducted a qualitative study using an ad hoc interview to analyze guilt feelings related to potential inconsistent work, using the Jacobsen Framework (a theoretical model for studying aesthetics psychology) to understand the effects of intrinsic and extrinsic motivation on CC self-esteem.

Yeshua-Katz and Martins [27] also used a qualitative methodology with a focus on personal narratives and the meanings individuals assign to their experiences with illness. In addition, content analysis based on grounded theory was used to identify emerging themes.

Ressler et al [28] performed a mixed methods study. They conducted a quantitative part with closed-ended questions to collect data on participants’ characteristics, using descriptive statistics. They also conducted a qualitative portion with open-ended questions to explore bloggers’ personal experiences, motivations, and perceptions of blogging’s impact on their emotional and social well-being, using grounded theory to identify recurring themes and patterns.

### Main Results

The study by Zsila et al [23] revealed results indicating that the lack of group belonging, along with certain types of video games, led to the development of mental health symptoms, such as anxiety and depression, as well as disturbances in sleep.

Niederauer and Maggi [24] highlighted how the lack of satisfaction in content creation could lead to signs and symptoms of burnout. They observed that unsatisfactory CC on YouTube led to various health problems, including exhaustion, discomfort, pressure, distress, anxiety, and burnout.

In the study conducted by Gandhi et al [25], it was described that the type of video game influenced how mental health was addressed for both CCs and their audiences, without specifying concrete symptoms.

The results of the study by Tran et al [26] indicated signs or symptoms of burnout in the studied population. Feelings of guilt related to unconscious work, delayed video posts, artistic blocks, and failure to keep social media platforms up to date were observed. In addition, mood regulation, self-esteem, and anxiety were reflected.

Yeshua-Katz and Martins [27] highlighted the presence of bloggers with health concerns related to eating disorders, manifesting issues such as anorexia nervosa and anxiety.

Ressler et al [28] showed that content creation for bloggers dealing with chronic pain and illness helped reduce the feeling of isolation through online communication, presenting a therapeutic potential in the act of blogging.

Thus, both Yeshua-Katz and Martins [27] and Ressler et al [28] suggested that blogging could serve as an effective means to express a patient’s clinical situation and even be considered a therapeutic activity.

All studies agreed on the need for further research into the potential pathological processes of CCs engaged in social media activities.

## Discussion

### Principal Findings

Considering the recommendations for the objectives of a systematic review [29], this review summarized relevant information related to the occupational health of CCs working on social media. In fact, there is a wide range of research on the effects of social media use on health, particularly in young people. However, very little is known about the health of social media CCs, who also tend to be quite young.

The reviewed papers presented low obsolescence, indicating that the topic is novel. This is further confirmed by the high percentage of papers included in this study that were published in the last 5 years, ensuring confidence in the findings being up to date.

The evaluation of the document accuracy of the studies included in this review, using the STROBE guidelines, did not reveal any temporal evolution. Usually, more recent papers show better results and are linked to the progressive implementation of quality questionnaires. In fact, older studies often did not follow these quality guidelines; for example, the first documents in STROBE date back to 2004, and their usage was gradual [30]. In light of the recent publication of the reviewed studies, it was anticipated that this circumstance would not arise and that the obtained results would be satisfactory.

The level of evidence and recommendation grade for this work, according to the SIGN criteria, were in line with expectations for reviewing observational studies. Despite aiming for a consistent relationship between intervention and outcome, it is important to remember the study designs from which the evidence is derived, acknowledging that some are more susceptible to biases than others and, therefore, justify decisions less strongly. It is well known that many health and occupational safety studies still do not rely on the highest possible level of evidence [31]. This could be due to the limitations of certain primary study designs, such as clinical trials, which are considered robust but may not be suitable for evaluating occupational health interventions, as they generally have long-term effects [32]. In this review, the pathological processes of CCs may not yet be well-studied causes.

The evaluation of the domains included in the ROBINS-E tool showed the main expected biases in the observational studies, but it should be noted that no studies with appropriate cause-and-effect relationships were available, as already indicated [21]. In fact, studies on occupational health, particularly those analyzing data from social media, have shown an uncontrolled process that leads to a biased focal point [33].

On the other hand, the population size was very small, except for the studies by Zsila et al [23] and Ressler et al [28], which could establish results directly derived from the intervention and, consequently, generalize their conclusions. This issue was mentioned in all studies, which unanimously agreed on the need for further research into the possible pathological processes of CCs engaged in social media activities.

Regarding the activity developed, it was observed over the years that there has been an evolution from blogging to the use of platforms that allow live streaming, such as YouTube or Twitch, as streamers can broadcast in real time and interact with their audience. Despite the differences each platform may present, there is a unique characteristic: the effort of CCs to attract audiences and establish parasocial relationships (ie, emotional connection with someone they do not know personally) [34]. Furthermore, people aged 15-24 years (the most active on social media) are precisely those who use platforms such as YouTube and Twitch the most [35]. These 2 platforms have contributed to the so-called “democratization of content generation,” allowing the audience to have a proactive role in the process. The “Twitch Generation” values, above all, authenticity and interactivity, genuine, unfiltered content, and direct interactions with their favorite streamers [36].

The presence of minors as CCs in the study by Yeshua-Katz and Martins [27] should not come as a surprise, as most platforms allow content creation starting at the age of 13. However, we must not forget that these minors are performing adult-level work, with all the associated risks, particularly from a psychological perspective. Therefore, it is crucial to raise awareness among parents and guardians while also keeping in mind the responsibility of digital platforms in safeguarding minors’ rights.

In this regard, the study by Gómez et al [37] examined how “kidfluencers” (minor CCs) created content and integrated “branded content” (content linked to a brand that connects the brand with the consumer) into their creations. Martínez Pastor et al [38] demonstrated that, despite the existence of advertising regulations in all the countries studied, compliance was minimal when it came to identifying advertising content as such. In this vein, Tur-Viñes et al [39] assumed that content creation by minor CCs is carried out with the support of parents, and in some cases, parents even open their own channels to support the transmedia strategy initiated by their children.

While the study by Ressler et al [28] included a population up to 75 years old, no specific results were found for the 65‐75 age range. However, other studies showed that the content production of senior CCs was characterized by a pronounced digital marketing strategy, highlighting professional routines such as regularity in posting frequency, post quality, and creativity, and a close relationship with followers [40].

Traditionally associated with a more passive role as content consumers, some older adults have been developing their digital skills and are now active CCs on social media. In some cases, they have even created a vast audience and a wide range of engaged, interested, and committed followers [41]. However, there is little evidence concerning these older digital CCs, and even less regarding the pathologies associated with this activity, as these individuals are often considered excluded from the labor market.

### Methodological Considerations

Conducting interviews online or via email are methods commonly used for data collection, as they are extremely cost-effective and can be sent to a large audience simultaneously. However, the reliability of the data can sometimes be questioned, as the responses received may not always be authentic. Therefore, a method should be established to support the results that might be obtained. Nevertheless, there are studies that have considered online or email interviews as an effective and convenient method [42].

In studies with quantitative methodology [23,28], a validated tool should have been used, or the homogeneity, reliability, internal consistency, and validity should have been reported [43]. In addition, in studies with qualitative techniques, the trustworthiness—credibility, transferability, dependability, and confirmability—should have been discussed [44].

The need for validation of results is fundamental in web-based studies. Collecting high-quality datasets from social media users is problematic, not only due to the biases associated with data collection methods but also with respect to consent management and the selection of appropriate analytical techniques [45].

### Health Impacts and Preventive Measures

As observed in this review, the impact on mental health was one of the primary issues resulting from the activities of CCs. This is due to the constant pressure, whether imposed externally or self-imposed, that these workers face in their daily activities to meet targets.

At the same time, they must contend with the need to please their viewers, often unable to fully express their emotions or feelings, either to their audience or, even less so, to their sponsors. This situation leads to the manifestation of symptoms that affect the mental health of CCs, such as disturbances in sleep, anxiety, emotional lability, excessive worry, irritability, loss of interest, obsessions, and fatigue. This set of symptoms creates the ideal breeding ground for the development of psychiatric illnesses such as generalized anxiety disorder, dysthymia, or depressive episodes. In the most extreme cases, these can even lead to the suicide of a CC [46,47].

In addition to the extreme burnout associated with these conditions, CCs may also experience physical exhaustion and a sense of depersonalization, resulting in burnout syndrome, as indicated in the studies by Niederauer and Maggi [24] and Tran et al [26].

However, as demonstrated in this review, it would be a mistake to think that the health consequences for CCs are limited solely to mental health disorders. The prolonged use of data display screens, visual fatigue, persistent forced postures, repetitive upper limb movements, and a sedentary lifestyle can lead to musculoskeletal disorders and systemic issues. These risks associated with the occupational activity of CCs are critical to understanding the various impacts that this professional activity can have on the health of these workers.

It is essential to address these consequences at their source through preventive actions to avert health problems. To prevent these health issues, CCs should be educated and informed about the potential consequences of their work activity, emphasizing the importance of a balance between work and rest. Promoting periodic digital disconnection and avoiding marathon workdays that hinder recovery or rest is key. In this regard, it is important to identify whether CCs, or certain profiles within this group, are exposed to shift work in their activities and, if so, to apply the corresponding preventive measures. To ensure the health of workers, periodic evaluations of their well-being are essential and should be conducted regularly to identify and prevent potential health issues.

Regarding the consequences of sedentary behavior in CCs’ activities, workers must be made aware of the importance of taking regular breaks during their workday, alternating between sitting and standing, to reduce the negative effects of prolonged sedentary behavior.

To mitigate the risks associated with the use of data display screens, persistent forced postures, and repetitive upper limb movements, it is crucial for CCs to ensure adequate ergonomic conditions in their work environment that promote a healthy and appropriate setting for carrying out their activities.

### Critical Reflection by Authors

It is essential to consider the importance of conducting research that analyzes the impact on the health of CCs from multiple perspectives, including age, the type of Web 2.0 platform used for content creation, the target audience, the social impact generated, and the consequences for health in each of these areas. This multidimensional approach would allow for a comprehensive and detailed understanding of the occupational risks associated with this activity for the health of those engaged in it. The foundation of occupational risk prevention lies in addressing the problem at its origin. Furthermore, appropriate corrective and preventive measures must be established to minimize the risk. In this regard, there is a noticeable gap in proposals related to such measures. As long as the active population continues their work without regular medical supervision and appropriate follow-up, health problems will persist or develop, which, if left uncorrected, could lead to severe and irreversible damage to the CCs.

### Limitations

A primary limitation of this review could be the low number of studies selected, as it concerns an emerging area of knowledge. It has been stated that systematic reviews should be based on studies with designs and selections that ensure greater scientific rigor. However, this analysis included all relevant studies retrieved on the topic under investigation.

The significant difference between the papers retrieved and the final selection is primarily due to the bibliographic databases Scopus and Web of Science. Although some studies were retrieved from other databases, the majority were irrelevant, which could be attributed to their lack of proper indexing (the search was conducted in text format, consulting titles, abstracts, and keywords) and the inability to limit the search by paper type. This high “documentary noise” has been previously observed in other reviews.

According to the United States Agency for Healthcare Research and Quality, the epidemiological designs of the studies selected in this review do not guarantee the full validity and reliability of the observations obtained [48]. However, the available evidence is likely the best given the difficulties of studying this area of research and based on the observations obtained in the various interventions.

Although the real limitations are due to the characteristics of each study, important lessons can be drawn from them to formulate appropriate actions for the development, implementation, and evaluation of future Web 2.0 applications. As Drucker et al [49] rightly stated, understanding these sources of bias is crucial for both authors and consumers of scientific literature as they conduct and read systematic reviews and incorporate their findings into clinical practice and policy formulation.

### Conclusions

The studies revealed that the most observed area was mental health. Although the impact on mental health has been extensively documented, it is evident that there is a need to address other aspects of this occupational activity, such as physical health and its social impact, in future studies.

Content creation, therefore, is an emerging activity that is not without health issues, especially in the realm of mental health. It is crucial to continue conducting research that addresses these problems at their origin and determines preventive measures to mitigate their effects. Thus, more detailed studies from an occupational health perspective are necessary.

## Supplementary material

10.2196/76708 Checklist 1 PRISMA-ScR checklist.

## Abbreviations

BK: Burton-Kebler
CC: content creator
PI: Price Index
PRISMA: Preferred Reporting Items for Systematic Reviews and Meta-Analyses
PRISMA-ScR: Preferred Reporting Items for Systematic Reviews and Meta-Analyses extension for Scoping Reviews
ROBINS-E: Risk of Bias in Non-randomized Studies of Exposures
SIGN: Scottish Intercollegiate Guidelines Network
STROBE: Strengthening the Reporting of Observational Studies in Epidemiology
